# Inflammation, Haemostatic Disturbance, and Obesity: Possible Link to Pathogenesis of Diabetic Retinopathy in Type 2 Diabetes

**DOI:** 10.1155/2013/818671

**Published:** 2013-12-02

**Authors:** Martina Tomić, Spomenka Ljubić, Snježana Kaštelan, Antonela Gverović Antunica, Anamarija Jazbec, Tamara Poljičanin

**Affiliations:** ^1^Department of Ophthalmology, University Clinic Vuk Vrhovac, Clinical Hospital Merkur, Zajčeva 19, 10000 Zagreb, Croatia; ^2^Department of Endocrinology and Metabolic Diseases, University Clinic Vuk Vrhovac, Clinical Hospital Merkur, Zajčeva 19, 10000 Zagreb, Croatia; ^3^Department of Ophthalmology, Clinical Hospital Dubrava, Avenija Gojka Šuška 6, 10000 Zagreb, Croatia; ^4^Department of Ophthalmology, General Hospital Dubrovnik, Dr. Roka Mišetića 2, 20000 Dubrovnik, Croatia; ^5^Faculty of Forestry, University of Zagreb, Svetošimunska 25 p.p. 422, 10002 Zagreb, Croatia; ^6^Croatian National Institute of Public Health, Rockefellerova 7, 10000 Zagreb, Croatia

## Abstract

*Purpose.* The pathogenesis of diabetic retinopathy (DR) is insufficiently understood but may possibly involve chronic, low-grade inflammation. The aim of this cross-sectional study was to investigate the relationship between inflammatory and haemostatic markers, other markers of endothelial dysfunction and anthropometric parameters, and their association with DR in patients with type 2 diabetes. *Methods.* According to the DR status patients were divided into three groups: no retinopathy, mild/moderate nonproliferative (NPDR), and severe NPDR/proliferative retinopathy (PDR). *Results.* The groups did not differ in the levels of inflammatory and haemostatic markers, other markers of endothelial dysfunction, and anthropometric parameters. After dividing the patients according to the level of obesity (defined by BMI, WC, and WHR) into three groups ANOVA showed the differences in C-reactive protein according to the WC (*P* = 0.0265) and in fibrinogen according to the WHR (*P* = 0.0102) as well as in total cholesterol (*P* = 0.0109) and triglycerides (*P* = 0.0133) according to the BMI. Logistic regression analyses showed that diabetes duration and prolonged poor glycemic control are the main predictors of retinopathy in patients with type 2 diabetes. *Conclusion.* Interrelations between obesity, inflammation, haemostatic disturbance, and other risk factors may possibly play an important additional role in endothelial dysfunction involved in the pathogenesis of diabetic retinopathy.

## 1. Introduction

Diabetes is the most frequent endocrine disease in developed countries and one of the most common noncommunicable diseases (NCDs) globally, estimated to have affected more than 371 million people in 2012 and projected to affect 552 million by 2030 [1]. It is the fourth or fifth leading cause of death worldwide with 4.8 million deaths in 2012, and its complications account for a significant portion of morbidity, mortality, and healthcare system cost burdens [1–3]. It is undoubtedly one of the most challenging health problems in the 21st century.

Diabetes has many manifestations in the eye, of which cataract and diabetic retinopathy are the most significant cause of visual impairment and blindness, and people with diabetes are 25 times more likely than the general population to become blind. Diabetic retinopathy (DR), a long-term microvascular and visually devastating diabetic complication, is estimated to be the leading cause of new blindness in working-aged adults in developed countries [4, 5]. Many epidemiological and clinical trials have proven the impact of diabetes duration, poor glycemic control, and hypertension on the prevalence, incidence, and progression of diabetic retinopathy [6, 7]. Although these factors explain a significant portion of the presence and progression of retinopathy and of the incidence of proliferative retinopathy [8], the exact pathogenesis of diabetic retinopathy is still insufficiently understood. Dysfunction of retinal endothelium is thought to be a possible mechanism as it plays a crucial role in all stages of diabetic retinopathy [9, 10]. Strategically located between blood and tissue, healthy endothelium actively regulates vascular tone and permeability, the balance between coagulation and fibrinolysis, the composition of the subendothelial matrix, the extravasation of leukocytes, and the proliferation of vascular smooth muscle. To perform these functions, endothelium produces components of the extracellular matrix and a variety of regulatory mediators. Functional impairment of endothelial activity precedes the development of morphological alterations during the progression of diabetes and its vascular complications. This endothelial dysfunction results from reduced bioavailability of the vascular nitric oxide (NO), mainly due to accelerated NO degradation by reactive oxygen species (ROS). Although hyperglycemia, insulin resistance, hyperinsulinemia, and hyperlipidemia independently and/or simultaneously contribute to endothelial dysfunction via several different mechanisms [11], systemic inflammation and hemorheological alterations found in obese diabetic patients may possibly play an important role in the endothelial dysfunction and in the etiopathogenesis of diabetic retinopathy [12, 13].

Many studies have documented the association of inflammation, haemostatic disturbance, and endothelial dysfunction with macroangiopathy in obese nondiabetic individuals and type 2 diabetic patients [14, 15], but only some of them have investigated the association of inflammation and endothelial dysfunction with the prevalence and progression of diabetic microangiopathy [16, 17].

The aim of the present study was to investigate the relationship between inflammatory and haemostatic markers, other markers of endothelial dysfunction and anthropometric parameters, and their association with diabetic retinopathy in patients with type 2 diabetes.

## 2. Patients and Methods

This cross-sectional study was performed in collaboration between the Department of Endocrinology and Metabolic Diseases and the Department of Ophthalmology of the University Clinic Vuk Vrhovac Clinical Hospital Merkur in Zagreb in accordance with the Declaration of Helsinki and approved by the Hospital's Ethics Committee. The patients included in the study received both written and oral information about the study and signed a written informed consent.

### 2.1. Patients

A total of 107 patients with type 2 diabetes consecutively attending both departments over a six-month period were included in the study. They were on either oral hypoglycemic agent (OHA) therapy or insulin therapy. Type 2 diabetes was defined according to the American Diabetes Association classification [18]. Patients with malignancies, immunologic, infectious inflammatory diseases, patients receiving corticosteroids or cytostatics, pregnant women, and patients with other eye diseases (mature cataract, uveitis, and age-related macular degeneration) were not included in the study.

### 2.2. Methods

Patients who met all inclusion criteria were invited to participate in the study. At the inclusion visit, the informed consent form was signed, blood samples for laboratory analyses were collected between 08:00 and 10:00 h after 12 h overnight fast, and complete clinical and ophthalmic examination was performed.

#### 2.2.1. Marker of Inflammation

C-reactive protein (CRP) was determined by an automated immunoturbidimetric assay on an Olympus AU600 analyzer (Olympus Optical Co., Tokyo, Japan) (reference value < 5.0 mg/L) [19].

#### 2.2.2. Marker of Haemostatic Disturbance

Fibrinogen was measured by the Clauss method (reference values 1.8–4.1 g/L) [20].

#### 2.2.3. Other Markers of Endothelial Dysfunction

Glycated hemoglobin value (HbA_1_c), total cholesterol, HDL cholesterol, LDL cholesterol, and triglycerides were measured. HbA_1_c was determined at the beginning of the study from a single venous blood sample, and HbA_1_c_median_ was obtained by statistical analysis of data from the National Registry for Diabetes (CroDiabNet). The statistical analysis included HbA_1_c values from venous blood samples taken from each individual patient at 3-4-month intervals over the past three years. HbA_1_c was determined by an automated immunoturbidimetric assay (reference values 3.5–5.7%) [21]. Total cholesterol and triglycerides were measured by the enzymatic colorimetric tests (reference values: total cholesterol < 5.00 mmol/L; triglycerides < 1.70 mmol/L) [22, 23].

#### 2.2.4. Anthropometric Parameters

Body mass index (BMI) as a common index of obesity was calculated by dividing weight and height squared (kg/m^2^). Weight was measured using a balance-beam scale and height was measured using a wall-mounted stadiometer with patients in their underwear and without shoes. Recommended value among men was considered <23 and among women < 22 kg/m^2^ with a normal range between 18.5 and 24.9 kg/m^2^ [24]. Waist circumference (WC), a direct indicator of abdominal obesity, was measured in the middle distance between the last floating rib and the iliac crest (cm). Recommended values were considered <94 cm (men) and <80 cm (women) [25]. The waist-to-hip ratio (WHR) as an index of body fat distribution was determined by dividing waist and hip circumference. The hip circumference was measured with a measuring tape passing on femoral trochanters (cm). Suggested values of WHR were considered as <1.0 (men) and <0.8 (women) [25].

#### 2.2.5. Clinical Parameters

Blood pressure was measured with an ambulatory sphygmomanometric device after a 5 min rest, and a mean of three measurements was used. Hypertension was defined as blood pressure >130/80 mmHg.

#### 2.2.6. Ophthalmologic Examination

Complete eye examination included best corrected visual acuity (BCVA), Goldmann applanation tonometry, slit lamp biomicroscopy of the anterior eye segment, binocular indirect slit lamp fundoscopy, and fundus photography after mydriasis with eye drops containing 0.5% tropicamide and 5% phenylephrine. Color fundus photographs of two fields (macular field, disc/nasal field) of both eyes were taken with a suitable 45° fundus camera (VISUCAM, Zeiss) according to the EURODIAB retinal photography methodology [26]: macular field: positioned in such a way that the exact center of the optic disc is laid at the nasal end of the horizontal meridian of the field view; disc/nasal field: such that the optic disc was positioned one disc-diameter in from the temporal edge of the field, on the horizontal meridian. EURODIAB classification scheme was used because it uses two-field 45° fundus photography and standard photographs to grade retinal lesions [26]. In each patient the eye more affected was graded for diabetic retinopathy using fundus photographs. Modified Scheie classification of hypertension retinopathy and classification of hypertension retinopathy by Wong and Mitchell were used to categorize the retinal vascular changes caused by hypertension [27, 28].

### 2.3. Statistical Analyses

For all analyzed variables descriptive statistics (*n*, mean ± standard deviation, percentages) were used. In all analyses *P* value of less than 0.05 was considered statistically significant. Differences in distributions of continuous data were determined by ANOVA or Kruskal-Wallis test. Differences in distributions of categorical data were evaluated by Chi-square test. The normality of distribution was tested by Shapiro-Wilks *W* test and homogeneity of variance by Leven test. To compare analyzed variables (C-reactive protein, fibrinogen, HbA_1_c_median_, total cholesterol, and triglycerides) according to the diabetic retinopathy status and the level of obesity (defined by BMI, WC, and WHR), ANOVA with two main factors and their interaction was used [29]. Univariate and multiple logistic regression analyses were used to assess the strength and independence of associations. All analyses and graphics were performed using STATISTICA 12.0. [30].

## 3. Results

This study included 107 patients with type 2 diabetes (67 male, 40 female) with a mean age 66.74 ± 8.01 years and a mean diabetes duration of 15.05 ± 5.69 years. Forty (37%) patients were on oral hypoglycemic agents (OHA) and 67 (63%) on insulin therapy.

The average best corrected visual acuity (BCVA) of our patients was 0.91 ± 0.22, and the average intraocular pressure (IOP) was 13.55 ± 1.33 mmHg. Nine (8%) patients were suffering from primary open angle glaucoma (POAG) and were treated with topical antiglaucomatous therapy. 16 (15%) patients had clear crystalline lenses, 74 (69%) an initial cataract, and 17 (16%) patients had the condition after cataract surgery (an artificial IOL implanted). Hypertensive retinopathy was detected in 40 (37%) patients.

According to the two-field 45° color fundus photography (EURODIAB standards) [26] patients were divided into three groups: DR group 1—patients with no retinopathy (*n* = 65), DR group 2—patients with mild/moderate nonproliferative diabetic retinopathy (NPDR; *n* = 19), and DR group 3—patients with severe/very severe NPDR or proliferative diabetic retinopathy (PDR; *n* = 23).

Ophthalmologic parameters of type 2 diabetic patients divided according to the diabetic retinopathy status are presented in Table 1. DR group 3 was found to have significantly lower best corrected visual acuity (BCVA) than DR group 1 (0.72 ± 0.37 versus 0.97 ± 0.08; *P* = 0.001). The presence of cataract as well as the condition after cataract surgery (an artificial IOL implanted) was observed significantly more often in DR groups 2 and 3 than in DR group 1 (*P* = 0.023). Hypertensive retinopathy was observed as marginally significant more often in DR groups 2 and 3 than in DR group 1 (*P* = 0.079).

Table 2 presents descriptive statistics of basic characteristics, inflammatory and haemostatic markers, other markers of endothelial dysfunction, and anthropometric and clinical parameters of type 2 diabetic patients divided according to the diabetic retinopathy status. DR group 3 had significantly longer duration of diabetes (19.35 ± 4.60 years versus 13.22 ± 5.08 years; *P* < 0.001) and more often insulin than OHA therapy (87/13% versus 52/48%; *P* = 0.009) in comparison with DR group 1. The three groups did not significantly differ in the levels of inflammatory and haemostatic markers, other markers of endothelial dysfunction, and anthropometric and clinical parameters, with the exception of marginally significant difference in HbA_1_c_median_ between the DR group 3 and DR group 1 (7.31 ± 0.85 versus 6.77 ± 0.76; *P* = 0.055).

To investigate the specific relationship between the inflammatory and haemostatic markers, other markers of endothelial dysfunction, diabetic retinopathy, and obesity, patients were additionally divided according to the anthropometric parameters (BMI; body mass index, WC; waist circumference, WHR waist-to-hip ratio,) [24, 25] into three groups. BMI groups are BMI group 1—patients with BMI ≤ 25 kg/m² (*n* = 10), BMI group 2—patients with BMI 26–29.9 kg/m² (*n* = 48), and BMI group 3—patients with BMI ≥ 30 kg/m² (*n* = 49). WC groups are WC group 1—patients with WC ≤ 94 (m) or ≤80 (w) cm (*n* = 11), WC group 2—patients with WC 95–110 (m) or 81–95 (w) cm (*n* = 37), and WC group 3—patients with WC ≥111 (m) or ≥96 (w) cm (*n* = 59). WHR groups are WHR group 1—patients with WHR ≤ 1.0 (m) or ≤0.8 (w) (*n* = 16), WHR group 2—patients with WHR 1.01–1.1 (m) or 0.81–0.9 (w) (*n* = 47), and WHR group 3—patients with WHR ≥1.11 (m) or ≥0.91 (w) (*n* = 44).

**Figure 1:**
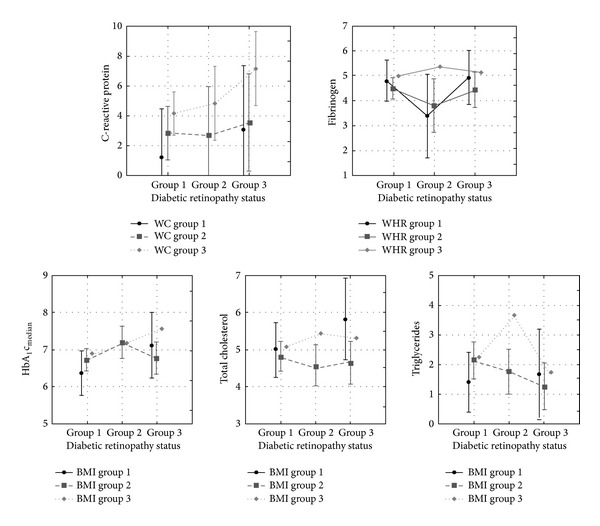
Vertical bars denote 0.95 confidence intervals. BMI: body mass index (kg/m²); WC: waist circumference (cm); WHR: waist-to-hip ratio. Statistically significant differences in C-reactive protein, fibrinogen, HbA_1_c_median_, total cholesterol, and triglycerides in type 2 diabetic patients divided into three groups according to the diabetic retinopathy status and the level of obesity defined by BMI, WC, and WHR.

Table 3 presents the differences in C-reactive protein, fibrinogen, HbA_1_c_median_, total cholesterol, and triglycerides in type 2 diabetic patients divided into three groups according to the diabetic retinopathy status and the level of obesity (defined by BMI, WC, and WHR). The statistically significant difference in CRP was observed according to the level of WC (*P* = 0.0265), while no significant differences in CRP were observed according to the DR status, level of BMI and WHR, or the interaction between the DR status and the level of obesity. The statistically significant difference in fibrinogen was found according to the level of WHR (*P* = 0.0102), while no significant differences in fibrinogen were found according to the DR status, level of BMI and WC, or the interaction between the DR status and the level of obesity. The significant difference in HbA_1_c_median_ was observed according to the DR status (*P* = 0.0312), while there were no significant differences according to the level of obesity or interaction of DR status and the level of obesity. The significant differences in total cholesterol and triglycerides were observed only according to the level of BMI (total cholesterol *P* = 0.0109; triglycerides *P* = 0.0133), while there were no significant differences according to the DR status, level of WC and WHR, or interaction between DR status and the level of obesity. Statistically significant differences observed by ANOVA with two main factors and their interaction are presented in Figure 1.

Univariate and multiple logistic regression analyses showed that diabetes duration, insulin therapy, and prolonged poor glycemic control (HbA_1_c_median_) were the main predictors of retinopathy in patients with type 2 diabetes (Table 4). The increasing prevalence of retinopathy was significantly associated with longer duration of diabetes (OR = 1.17, 95% CI 1.08–1.27), even after adjustment for age and sex (AOR = 1.2, 95% CI 1.1–1.32). Insulin therapy, not obligatory in type 2 diabetes but often used in patients with prolonged diabetes duration and poor glycemic control, increased the prevalence of retinopathy to a significant extent (OR = 3.34, 95% CI 1.38–8.09), even after adjustment for age and sex (AOR = 3.28, 95% CI 1.34–8.02). The increasing prevalence of retinopathy was significantly related to prolonged poor glycemic control (HbA_1_c_median_) (OR = 1.76, 95% CI 1.08–2.86), being equal after adjustment for age and sex (AOR = 1.84, 95% CI 1.10–3.06).

## 4. Discussion 

Due to its high prevalence, incidence, and risk of macrovascular and microvascular diabetic complications, type 2 diabetes is one of the potentially most damaging diseases and biggest public health problems at the present time. Diabetic eye disease with its complications, especially diabetic retinopathy which leads to macular edema and retinal neovascularization, is the leading cause of visual dysfunction and blindness among working-aged adults in economically developed societies worldwide.

As expected, we found a significantly lower visual function, defined by best corrected visual acuity (BCVA), in the group of patients with severe NPDR and PDR compared to the group of patients with no retinopathy. Also, a significantly more frequent cataract and the condition after cataract surgery (an artificial IOL implanted) were observed in the groups of patients with diabetic retinopathy (NPDR and PDR) as compared to the group of patients with no retinopathy. Some previous studies have found similar results [31, 32]. Pollreisz and Schmidt-Erfurth in their review attribute this to the activation of the polyol pathway by intracellular hyperglycemia, which leads to the sorbitol-induced osmotic stress and occurrence of a typical snowflake diabetic cataract or earlier senile cataract [32].

The results of logistic regression analyses in our study are supported by many previous epidemiological and clinical studies, suggesting that diabetes duration and prolonged poor glycemic control are the main predictors of the prevalence and progression of retinopathy in patients with type 2 diabetes [5, 33, 34]. We also found insulin therapy to be among the main predictors of retinopathy in this type of diabetes. It is common knowledge that insulin therapy is not obligatory for patients with type 2 diabetes, but in these patients it is often necessary in progressive insulinopenia, prolonged duration of diabetes, and very poor glycemic control. Our results are similar to those of the Wisconsin Epidemiologic Study of Diabetic Retinopathy, which reported an increased 4- and 10-year cumulative incidence and significantly increased prevalence of diabetic retinopathy in older patients on insulin therapy than those on OHA therapy (70% versus 39%) [6].

Hypertension is another risk factor for development and progression of diabetic retinopathy documented in many epidemiological and clinical studies. Two large clinical studies, the United Kingdom prospective diabetes study (UKPDS) and the appropriate blood pressure control in diabetes (ABCD) study, demonstrated benefit in retinopathy risk reduction in diabetic patients who received intensive blood pressure control therapy [35, 36]. On the basis of data from these studies, clinical guidelines now recommend optimization of blood pressure control in type 2 diabetic patients as part of preventive measures for visual loss due to diabetic retinopathy. In our study there was no significant difference in the level of systolic and diastolic blood pressure between the groups according to their diabetic retinopathy status. It is worth noting that the average systolic blood pressure among our patients was 142.34 ± 23.03 mmHg and the average diastolic blood pressure was 81.07 ± 12.53 mmHg, these values being very near to those recommended by the American and European Societies of Cardiology [37, 38]. Numerous studies reported that early diabetic and hypertensive retinopathy signs share a number of similar morphological features representing small vessel damage [39, 40]. One challenge that often confronts clinicians is to understand the contribution of diabetes or hypertension in the development of retinopathy. An acute increase in blood pressure may cause retinal vascular changes that are very similar to the retinal vascular lesions seen in mild and/or moderate nonproliferative diabetic retinopathy. Although similar, some clinical signs are more specific showing distinct morphological differences. For example, retinal arteriolar abnormalities, such as generalized or focal arteriolar narrowing and arteriovenous nicking, are commonly seen in patients with hypertension, whereas these arteriolar changes are not usually present in diabetic patients without hypertension [41, 42]. Clustering of microaneurysms may be a feature pointing more towards diabetes and has been shown to predict diabetic retinopathy progression. Isolated retinal microaneurysms, on the contrary, may indicate hypertensive retinopathy in association with focal retinal arteriolar signs [42]. In clinical practice hypertension and diabetes frequently coexist and are known to result in more severe diabetic retinopathy [43]. This could partially explain some of our results. In spite of the near-normal average values of systolic and diastolic blood pressure among our patients, in the groups of patients with diabetic retinopathy (NPDR and PDR) we found marginally significant more often clinical sings of hypertensive retinopathy than in the group of patients with no retinopathy.

In addition to these well-known risk factors, new data suggest that adipose tissue is an important determinant of a low-level, chronic inflammatory state reflected by the production of various proinflammatory cytokines. These cytokines induce insulin resistance and endothelial dysfunction, consequently linking the later phenomenon with obesity and diabetic angiopathy [44–46]. Some studies have shown the correlation between obesity and diabetic retinopathy in patients with type 2 diabetes [34, 47, 48]. Moreover, growing data suggest that inflammation and hypercoagulable state are strongly related to the prevalence and progression of diabetic retinopathy [49–52]. van Hecke et al. in the Hoorn Study have found a positive association between the levels of C-reactive protein and soluble intracellular adhesion molecule-1 (sICAM-1) in the prevalence of diabetic retinopathy [16]. Nguyen et al. in the multiethnic study of atherosclerosis have observed the association of fibrinogen and plasmin-*α*2-antiplasmin complex (PAP) with any stage of diabetic retinopathy and PAP and homocysteine with vision-threatening diabetic retinopathy [51]. In our study there was no significant difference in the levels of inflammatory and haemostatic markers, other markers of endothelial dysfunction, and anthropometric parameters between the groups according to the diabetic retinopathy status, with the exception of marginally significant difference in HbA_1_c_median_ between the group of patients with severe NPDR and PDR and the group of patients with no retinopathy. The lack of significant difference in the levels of analyzed markers and parameters in our study may be a result of a relatively small sample size and due to the fact that the majority of our patients had near-normal values of these markers and parameters. However, we observed the significant difference in C-reactive protein according to the waist circumference and significant difference in fibrinogen according to the waist-to-hip ratio. Our results are similar to those of Nakamura et al., who reported that patients with metabolic syndrome had higher levels of C-reactive protein, and the main determinant of the CRP elevation was waist circumference [53]. García-Lorda et al. in their Mediterranean population study found C-reactive protein independently and positively associated to waist circumference and triglycerides and negatively associated to HDL-cholesterol [54]. C-reactive protein > 3 mg/L was found to be an independent risk factor for development of diabetic nephropathy and diabetic retinopathy [55], which confirmed the results of previously mentioned Hoorn study [16]. The multivariate analysis of the PRIME Study showed that waist-to-hip ratio, but not body mass index, was an independent predictor of fibrinogen [56]. These results were consistent with ours and results of two other cross-sectional studies suggesting that central body fat distribution is more relevant than general obesity to population correlates of fibrinogen [57, 58]. Cederholm-Williams et al. found higher plasma fibrinogen in diabetics than in controls and the highest fibrinogen in patients with more severe cases of retinopathy. As a direct consequence of the elevation of plasma fibrinogen they observed higher catabolic rate in diabetics than in controls and higher catabolic rate in patients with nonproliferative and proliferative retinopathy than in diabetics without retinopathy [59]. Fujisawa et al. suggested that an increased blood viscosity in type 2 diabetes patients due to high fibrinogen level and elevated intravessel pressure may play a role in the development of diabetic retinopathy [60]. In our study we also found the significant differences in total cholesterol and triglycerides according to the level of body mass index. Many previous epidemiologic studies have shown the association of body mass index and lipid profiles, especially higher total cholesterol and low-density lipoprotein cholesterol. Shamai et al. recently reported negative association of BMI with high-density lipoprotein cholesterol and positive association with triglycerides [61]. Investigating the potential risk factors for retinopathy in diabetic and nondiabetic individuals, the Hoorn study found positively associated prevalence of retinopathy with elevated blood pressure, BMI, total cholesterol, and triglyceride serum levels in all glucose categories [62]. Jew et al. suggested that HbA_1_c and total cholesterol are the two most important risk factors associated with clinically significant macular edema (CSME) in patients with nonproliferative diabetic retinopathy [63] whereas Zoppini et al. proposed that triglyceride/high-density lipoprotein cholesterol (TG/HDL-C) ratio is associated with an increased incidence of retinopathy and chronic kidney disease in patients with type 2 diabetes [64].

## 5. Conclusion

Diabetes duration, prolonged poor glycemic control, and the resulting need for insulin therapy are the main predictors of retinopathy in patients with type 2 diabetes. The significant differences in C-reactive protein, fibrinogen, total cholesterol, and triglycerides according to the level of obesity defined by different anthropometric parameters suggest that interrelations between obesity, inflammation, haemostatic disturbance, and other risk factors may possibly play an important additional role in endothelial dysfunction involved in the pathogenesis of diabetic retinopathy. Further studies that include larger number of patients and parameters such as inflammatory, haemostatic, and other markers of endothelial dysfunction are necessary to investigate whether our observations might contribute to the better understanding of the diabetic retinopathy causes and open new approaches for its prevention and treatment.

## Figures and Tables

**Table 1:** Ophthalmologic parameters of type 2 diabetic patients (*n* = 107) divided into three groups according to the diabetic retinopathy status.

	DR group 1 (*n* = 65)	DR group 2 (*n* = 19)	DR group 3 (*n* = 23)	*H* ^b^ Chi-square^c^	*P*
BCVA (decimal)*	0.97 ± 0.08	0.92 ± 0.15	0.72 ± 0.37	13.86^b^	0.001^b^
Glaucoma**	11	5	4	0.54^c^	0.461^c^
IOP (mmHg)*	13.66 ± 1.50	13.58 ± 1.07	13.22 ± 0.95	0.49^b^	0.487^b^
Lens**	20/71/9	5/69/26	9/65/26	7.5^c^	0.023^c^
Hypertensive retinopathy**	31	47	48	3.09^c^	0.079^c^

*mean ± SD, **percentage, ^b^Kruskal-Wallis df = 1, ^c^Chi-square test df = 2.

BCVA: best corrected visual acuity; IOP: intraocular pressure; Lens: clear crystalline lens/initial cataract/condition after cataract surgery (an artificial IOL implanted).

**Table 2:** Basic characteristics, inflammatory and haemostatic markers, other markers of endothelial dysfunction, and anthropometric and clinical parameters of type 2 diabetic patients (*n* = 107) divided into three groups according to the diabetic retinopathy status.

	DR group 1 (*n* = 65)	DR group 2 (*n* = 19)	DR group 3 (*n* = 23)	*F* ^a^ Chi-square^c^	*P*
Age (years)*	66.31 ± 8.31	68.47 ± 7.11	66.52 ± 7.98	0.543^a^	0.583^a^
Sex (m/f)**	65/35	42/58	74/26	0.28^c^	0.595^c^
Diabetes duration (years)*	13.22 ± 5.08	16.11 ± 6.01	19.35 ± 4.60	12.498^a^	0.001^a^
Therapy (OHA/insulin)**	48/52	32/68	13/87	7.52^c^	0.009^c^
C-reactive protein (CRP) (mg/L)*	3.37 ± 4.14	4.05 ± 3.34	5.36 ± 5.77	1.721^a^	0.184^a^
Fibrinogen (g/L)*	4.73 ± 1.23	4.75 ± 1.48	4.75 ± 1.09	0.002^a^	0.998^a^
HbA_1_c (%)*	6.42 ± 1.06	6.53 ± 1.06	6.70 ± 1.29	0.551^a^	0.578^a^
HbA_1_c_median_ (%)*	6.77 ± 0.76	7.18 ± 0.81	7.31 ± 0.85	2.976^a^	0.055^a^
Total cholesterol (mmol/L)*	4.96 ± 0.85	4.87 ± 1.27	5.05 ± 1.05	0.178^a^	0.838^a^
Triglycerides (mmol/L)*	2.14 ± 1.19	2.48 ± 2.41	1.50 ± 0.61	1.230^a^	0.066^a^
Body mass index (BMI) (kg/m²)*	30.77 ± 6.06	30.91 ± 5.28	30.12 ± 5.33	0.129^a ^	0.879^a ^
Waist circumference (WC) (cm)*	107.52 ± 14.96	108.21 ± 12.09	107.91 ± 12.28	0.020^a ^	0.980^a^
Waist-to-hip ratio (WHR)*	0.96 ± 0.08	0.96 ± 0.07	0.97 ± 0.07	0.162^a^	0.851^a^
Systolic blood pressure (mmHg)*	139.00 ± 22.97	151.32 ± 23.85	144.35 ± 21.18	2.267^a ^	0.109^a^
Diastolic blood pressure (mmHg)*	82.15 ± 12.90	80.26 ± 15.50	78.70 ± 8.15	0.691^a^	0.503^a^

*mean ± SD, **percentage, ^a^ANOVA df = 2, ^c^Chi-square test df = 1.

OHA: oral hypoglycemic agent; HbA_1_c: glycated hemoglobin value determined at the beginning of the study from a single venous blood sample; HbA_1_c_median_: glycated hemoglobin value obtained by statistical analysis of data from the National Registry for Diabetes (CroDiabNet).

**Table 3:** Differences in C-reactive protein, fibrinogen, HbA_1_c_median_, total cholesterol, and triglycerides in type 2 diabetic patients divided into three groups according to the diabetic retinopathy status and the level of obesity defined by BMI, WC, and WHR.

		CRP		Fibrinogen		HbA_1_c_median_		Total cholest.		Triglycerides	
	df	*F*	*P*	*F*	*P*	*F*	*P*	*F*	*P*	*F*	*P*
BMI 3 groups	1	0.660	0.4184	0.003	0.9588	2.877	0.0930	6.734	**0.0109**	6.353	**0.0133**
DR	1	1.711	0.1939	0.157	0.6927	4.776	**0.0312**	1.132	0.2899	0.986	0.3231
BMI 3 gr. ∗ DR	3	0.335	0.7997	0.878	0.4552	1.231	0.3025	0.938	0.4254	2.197	0.0932

WC 3 groups	1	5.077	**0.0265**	1.287	0.2593	0.763	0.3846	0.010	0.9217	0.676	0.4130
DR	1	2.395	0.1249	0.662	0.4180	3.479	0.0651	0.268	0.6057	0.891	0.3476
WC 3 gr. ∗ DR	3	0.320	0.8108	1.971	0.1233	0.142	0.9345	0.533	0.6604	1.063	0.3686

WHR 3 groups	2	2.536	0.0844	4.809	**0.0102**	0.790	0.4566	1.666	0.1943	1.697	0.1885
DR	2	2.163	0.1204	1.198	0.3063	2.875	0.0612	0.658	0.5200	1.795	0.1716
WHR 3 gr. ∗ DR	4	1.639	0.1704	1.061	0.3799	1.769	0.1411	0.341	0.8499	1.009	0.4065

DR: diabetic retinopathy; BMI: body mass index (kg/m²); WC: waist circumference (cm); WHR: waist-to-hip ratio; CRP: C-reactive protein; HbA_1_c_median_: glycated hemoglobin value obtained by statistical analysis of data from the National Registry for Diabetes (CroDiabNet).

**Table 4:** Odds ratios (95% CIs) for diabetic retinopathy associated with basic characteristics, inflammatory and haemostatic markers, other markers of endothelial dysfunction, and anthropometric and clinical parameters in type 2 diabetic patients (*n* = 107).

	OR	95% CI (OR)	AOR*	95% CI (AOR)
Diabetes duration (years)	**1.17**	1.08–1.27	**1.2**	1.1–1.32
Therapy (insulin)	**3.34**	1.38–8.09	**3.28**	1.34–8.02
C-reactive protein (CRP)	1.07	0.98–1.18	1.08	0.99–1.18
Fibrinogen	1.01	0.74–1.38	0.99	0.71–1.37
HbA_1_c	1.18	0.83–1.68	1.23	0.85–1.77
HbA_1_c_median_	**1.76**	1.08–2.86	**1.84**	1.10–3.06
Total cholesterol	1.01	0.68–1.51	1.03	0.68–1.57
Triglycerides	0.89	0.69–1.13	0.90	0.70–1.15
Body mass index (BMI)	0.99	0.93–1.06	0.99	0.92–1.07
Waist circumference (WC)	1.00	0.97–1.03	1.01	0.98–1.04
Waist-to-hip ratio (WHR)	1.34	0.01–185.05	7.42	0.14–3806.64
Systolic blood pressure	1.02	1.00–1.03	1.02	1.00–1.04
Diastolic blood pressure	0.98	0.95–1.01	0.98	0.95–1.02

Bold: statistically significant *α* = 0.05, *OR adjusted for age and sex.

HbA_1_c: glycated hemoglobin value determined at the beginning of the study from a single venous blood sample; HbA_1_c_median_: glycated hemoglobin value obtained by statistical analysis of data from the National Registry for Diabetes (CroDiabNet).
